# High sensitivity guided-mode-resonance optical sensor employing phase detection

**DOI:** 10.1038/s41598-017-07843-z

**Published:** 2017-08-08

**Authors:** Pankaj K. Sahoo, Swagato Sarkar, Joby Joseph

**Affiliations:** Photonics Research Lab, Department of Physics, Indian Institute of Technology Delhi, New Delhi, 110016 India

## Abstract

We report an ultra-sensitive refractive index (RI) sensor employing phase detection in a guided mode resonance (GMR) structure. By incorporating the GMR structure in to a Mach-Zehnder Interferometer, we measured the phase of GMR signal by calculating the amount of fringe shift. Since the phase of GMR signal varies rapidly around the resonance wavelength, the interference fringe pattern it forms with the reference signal becomes very sensitive to the surrounding RI change. The sensitivity comes out to be 0.608π phase shift per 10^−4^ RI change in water medium which is more than 100 times higher than the other reported GMR based phase detection method. In our setup, we can achieve a minimum phase shift of (1.94 × 10^−3^) π that corresponds to a RI change of 3.43 × 10^−7^, outperforming any of reported optical sensors and making it useful to detect RI changes in gaseous medium as well. We have developed a theoretical model to numerically estimate the phase shift of the GMR signal that predicts the experimental results very well. Our phase detection method comes out to be much more sensitive than the conventional GMR sensors based on wavelength or angle resolved scanning methods.

## Introduction

Guided Mode Resonance (GMR) is a resonance phenomenon where a diffracted order of grating excites a guided mode of a waveguide that again couples out of the waveguide and interferes with the zeroth order reflected light. Under certain phase matching conditions this interference produces a sharp peak in the reflection spectrum of the GMR device. This GMR peak by virtue of its narrow line width, high efficiency^1–3^ and sensitivity towards change in surrounding refractive index (RI) is widely accepted for bio-sensing^4, 5^ and many other applications^6–8^. Generally in case of GMR based biosensors, attaching a biolayer to the sensor grating surface changes the resonant peak wavelength which can be monitored using a spectrometer^9, 10^. The wavelength shift thus can be directly related to the change in RI due to addition of these biolayers and can be properly calibrated for detecting different biological samples. Apart from this wavelength scanning method; angular-resolved method that involves scanning through different incidence angles^5^ also enables sensing capability. However, both the wavelength and angle scanning detection methods operate through the mode of intensity detection whose sensitivity and limit of detection is limited by the low resolution of the spectrometers.

It is known that, the phase of the GMR signal varies rapidly around the resonance peak^11^. Thus sensors based on phase detection rather than wavelength or angle-resolved scan can be much more promising in terms of sensitivity and limit of detection. However, there are only very few reports on the GMR phase measurement and its application to RI sensors. Magnusson *et al*.^11^ theoretically showed a phase detection method by plotting a phase curve where they calculated the phase at each wavelength of the reflection spectrum using rigorous coupled wave theory. Kuo *et al*.^12^ have used the heterodyne interferometer configuration to measure the phase change of the GMR signal (taken as the resultant of s- & p- polarized light) with respect to a phase reference signal generated by a function generator. They have plotted the phase curve in terms of the incident angle and rotated an analyzer to tune this phase curve for higher sensitivity. They^13^ further used a five-step phase shift reconstruction algorithm^14^ to calculate the phase of the GMR signal at different incident angles and obtained the phase curve. They have used a liquid crystal retarder controller to vary the phase shift between s- & p-polarized components to be used in the algorithm. However, the aforementioned methods to detect the phase of the GMR signals are not straight forward and experimentally quite complex to be applicable for biosensors.

Here we report a simple, low cost and accurate method to measure the phase of a GMR signal using a Laser tuned Mach-Zehnder interferometer (MZI) configuration that does not involve any wavelength or angular scanning of phase as mentioned in the above cited works. The GMR signal is interfered with a reference signal to produce interference fringe pattern. By measuring the shift in the interference fringe pattern with respect to the change in surrounding RI, we obtained the sensitivity of our GMR device. We have developed a theoretical model to numerically calculate the phase of the GMR signal that matches very well with the experimentally measured phase change. This phase detection method turns out to be a very simple and efficient way to measure the phase of a GMR signal that can be applied to RI sensing and bio-sensing.

## Results and Discussions

**Figure 1:**
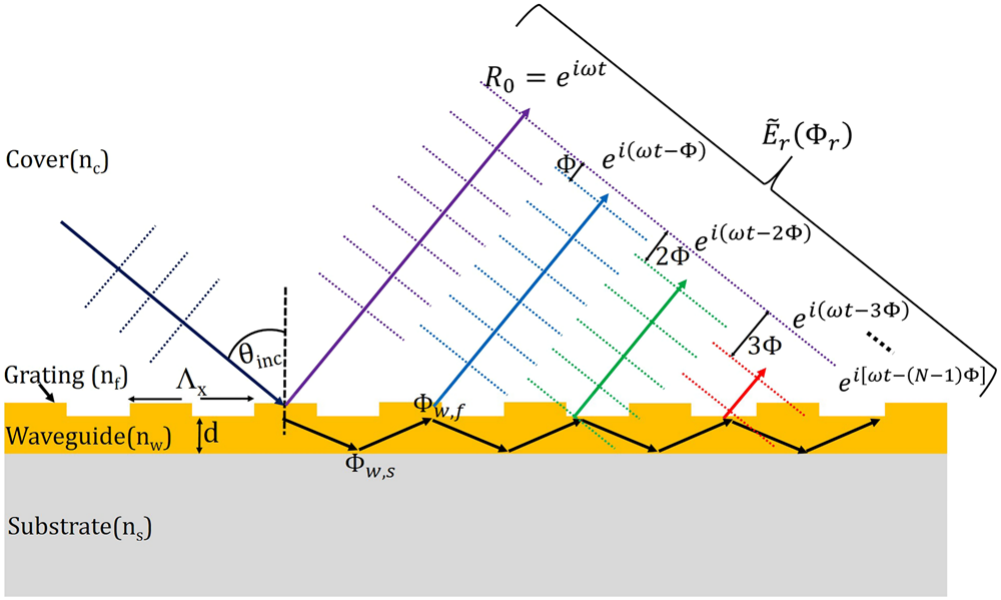
GMR structure explaining phase detection. A 1D subwavelength grating coupled to a waveguide to form the GMR structure. The change in the phase (Φ_*r*_) of the resultant complex wave (${\tilde{E}}_{r}$) is measured in response to surrounding RI change.

Consider a GMR structure as shown in Fig. 1. It consists of a 1D line grating of period ‘Λ_x_’ on a planar waveguide of thickness ‘d’ on a substrate. The RI of the cover, waveguide and substrate are denoted as n_c_, n_w_ & n_s_ respectively. The grating in our case is a subwavelength structure and can be considered as a uniform film of effective RI n_f_. A linearly polarized light is incident on the grating at an angle θ_inc_ as shown in the figure. After diffraction from the grating, the first order diffracted light travels inside the waveguide whose phase information^15, 16^ can be obtained from equation (1) as given below.

1 $${\rm{\Phi }}=2{k}_{c,w}d+{{\rm{\Phi }}}_{w,s}+{{\rm{\Phi }}}_{w,f}$$

where

2 $${k}_{c,w}={k}_{0}{({{n}_{w}}^{2}-{{n}_{eff}}^{2})}^{1/2}$$

and

3 $${n}_{eff}=\sqrt{{({n}_{inc}{\sin }{\theta }_{inc})}^{2}+2{n}_{inc}\,{\sin }\,{\theta }_{inc}\,{\cos }\,{\phi }_{inc}m\frac{{\lambda }_{0}}{\Lambda }+{m}^{2}\frac{{{\lambda }_{0}}^{2}}{{\Lambda }^{2}}}$$

The above expression of ‘n_*eff*_’ gives the effective index of the guided mode obtained from the phase matching condition^17, 18^ for the case of a GMR structure under conical diffraction^19, 20^ regime. The derivation of equation (3) is provided in the supplementary materials section. This expression of ‘n_*eff*_’ gives the dependency of the effective index on the basic waveguide-grating parameters such as Λ, λ_*0*_, θ_*inc*_ and the azimuthal angle ‘*φ*_*inc*_’. For φ_*inc*_ = 0°, equation (3) becomes,

4 $${n}_{eff}={n}_{inc}\,{\sin }\,{\theta }_{inc}+m\frac{{\lambda }_{0}}{\Lambda },$$

which is nothing but the ‘*n*_*eff*_’ for the case of general classical mount of GMR^21^. The first term (2*k*_*c,w*_ *d*) in equation (1) is the phase acquired due to optical distance traversed by the diffracted light while propagating inside the waveguide. Whereas Φ_*w,s*_ and Φ_*w,f*_ are the phase shifts due to total internal reflection at the waveguide-substrate and waveguide-film interface respectively whose dependency on the RI of various layers^15, 16, 22^ is given below.

5 $${{\rm{\Phi }}}_{w,s}=-2{\tan }^{-1}[{(\frac{{n}_{w}}{{n}_{s}})}^{2\rho }\frac{|{({{n}_{s}}^{2}-{{n}_{eff}}^{2})}^{1/2}|}{({{n}_{w}}^{2}-{{n}_{eff}}^{2})}]$$

6 $${{\rm{\Phi }}}_{w,f}=-2{\tan }^{-1}[{(\frac{{n}_{w}}{{n}_{f}})}^{2\rho }\frac{|{({{n}_{f}}^{2}-{{n}_{eff}}^{2})}^{1/2}|}{({{n}_{w}}^{2}-{{n}_{eff}}^{2})}]$$

Here *ρ* = 0 for TE & 1 for TM mode and *k*_0_ = 2*π*/*λ*_0_, with *λ*_0_ as the free space wavelength. While propagating along the waveguide, it gets diffracted by the grating each time it is incident on the grating interface. At each diffraction, the first order diffracted lights come out of the waveguide with a relative phase shift of Φ among themselves as shown in Fig. 1. Theoretically there are infinite number of such plane waves that are diffracted after each round trip inside the waveguide, whereas in the figure we have shown only the first three of them. All these diffracted waves interfere among themselves and also with the zeroth order reflected light (R_0_ = *e* ^*iωt*^) forming a resultant complex wave (${\tilde{E}}_{r}$) as in equation (7) below.

7 $${\tilde{E}}_{r}=\,{e}^{i\omega t}+{e}^{i(\omega t-{\rm{\Phi }})}+{e}^{i(\omega t-2{\rm{\Phi }})}+\ldots +{e}^{i[\omega t-(N-1){\rm{\Phi }}]}$$

**Figure 2:**
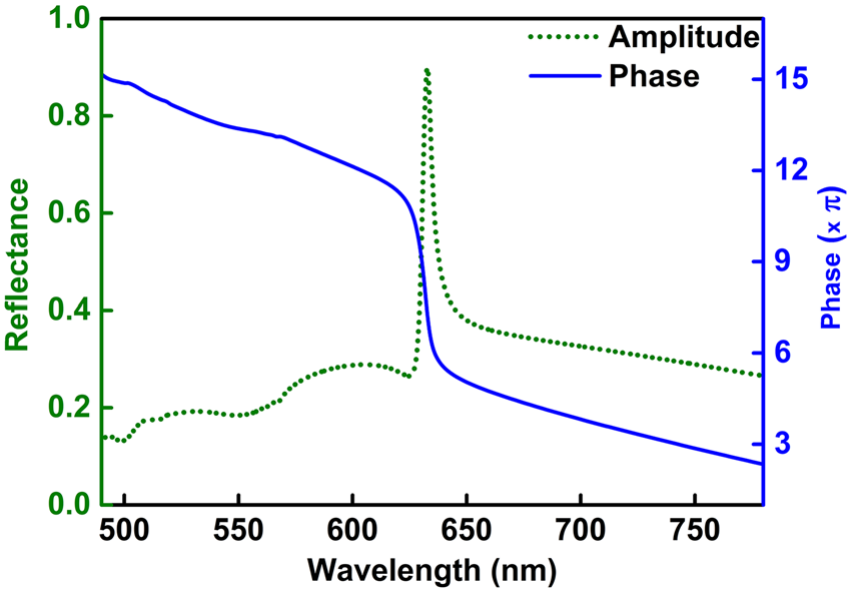
Phase curve of the GMR signal. Phase curve of the reflection spectrum of the GMR sample showing a rapid change in the phase value corresponding to the experimentally obtained resonance peak wavelength of 632.8 nm. Both amplitude reflectance and phase curves are shown. The curve has been produced using FDTD simulation tool.

This complex wave ${\tilde{E}}_{r}$ has a resultant phase Φ_*r*_ that depends on the relative phase difference Φ between all these diffracted waves. Under certain conditions, the relative phase change Φ between all these diffracted waves can become an integer multiple of 2*π* making all of them to be in phase. This implies that ${\tilde{E}}_{r}$ is now a plane wave with a resultant phase of Φ_*r*_ = 2 *mπ* (*m* = 0, 1, 2, …). Under this condition, there appears a sharp resonance peak at *λ*_0_ in the reflection spectrum known as GMR peak. Now, if there is a change in the RI of the cover region (n_c_), it leads to a change in the effective RI (*n*_*f*_) of the subwavelength grating layer (film) and the effective index (*n*_*eff*_) of the guided mode (following equation (3). All the above changes modify the three phase terms contained in equation 1. This change in Φ ultimately changes the resultant phase Φ_*r*_ of the complex wave ${\tilde{E}}_{r}$. It is this resultant phase Φ_*r*_ which we focus on in this paper and numerically calculated its value using equations (1–7). In our MZI setup, we measured the change in Φ_*r*_ (denoted by ∆Φ) by calculating the amount of fringe shifted in the fringe pattern formed due to the interference between GMR signal and reference signal. We performed the experiment with a GMR sample of the following parameters: silicon nitride (Si_3_N_4_ of RI ≈ 2.0) grating & waveguide layers of thickness 80 nm & 120 nm respectively on a quartz (RI ≈ 1.45) substrate, grating period is 500 nm with 50% duty cycle. In Fig. 2 we have shown the phase curve (blue solid curve) for the GMR sample having resonance peak at 632.8 nm. It can be seen from the figure that the phase of the curve changes rapidly around the GMR peak wavelength. Thus, a very small deviation from the resonance condition (due to the change in the surrounding RI) can induce a sharp jump in the phase of the GMR signal that makes the interference fringe pattern to shift significantly.

**Figure 3:**
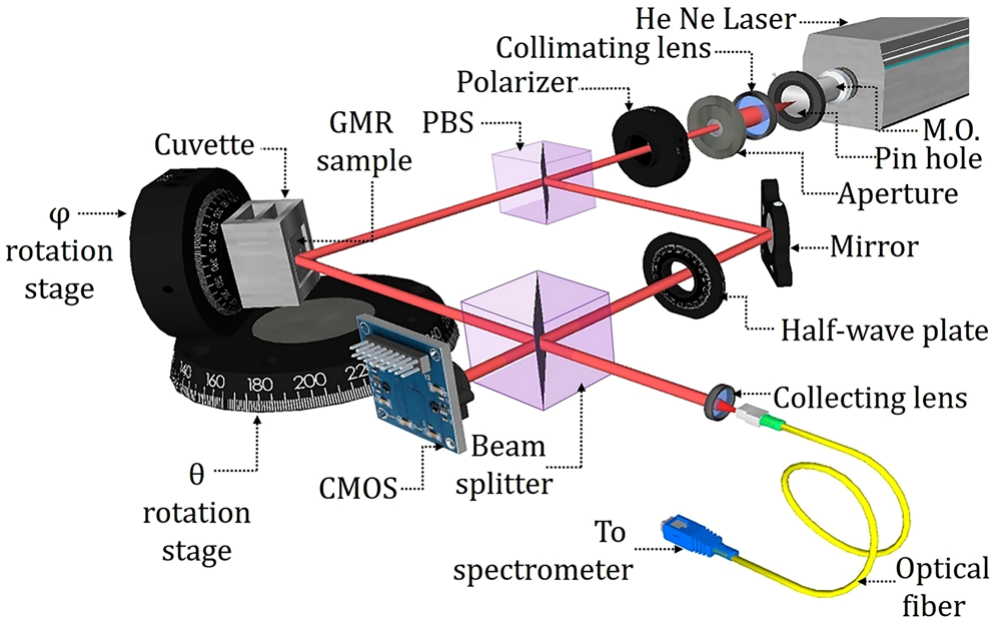
MZI setup. Schematic of the experimental setup of Mach Zehnder Interferometer to measure the phase shift of the GMR signal (drawn using Google SketchUp).

The experimental details to measure ∆Φ is discussed as follows, with a schematic (design procedure is given in the Methods section) of the experimental setup as shown in Fig. 3.We have used a He-Ne laser source (Spectra Physics Model 127) of wavelength 632.8 nm and 35 mW power for our experiment. The laser beam is divided in to two orthogonally linearly polarized beams by a polarizing beam splitter (PBS). One of them is incident on the GMR sample along one arm of the MZI called as the object arm and the other one passes through a mirror and a half wave plate (HWP) forming the reference beam. In our experiment we use DI water as the reference sensing medium having RI of 1.3352 and detect its RI change by addition of sucrose solution. Thus, before proceeding for the laser interference, we set the GMR position w.r.t. water medium and measured its reflection spectrum using a white light source (Avalight-DH-S-BAL). During this time the reference arm of the MZI is blocked. The reflected light is collected by a high NA lens and carried to the spectrometer (AvaSpec-ULS3648TEC) through a multimode optical fiber. The obtained spectrum shows a very sharp GMR peak at the desired wavelength of 632.8 nm. However, it is worthy to mention here that for practical application of our phase sensor, there is no need of any white light source or spectrometer to verify the resonance condition. The GMR condition can be easily set just by visualizing the intensity shootout of the laser beam at the exact resonance position. Since this method has not been carried out before, the white light source and spectrometer in our setup is used only to validate this fact. Although both the white light and laser source are used in our setup (Fig. S3 in supplementary section), due to the above reason we have not provided the white light source in the schematic diagram of the experimental setup in Fig. 3. It is also to be noted that, to tune the GMR peak wavelength exactly to our laser source wavelength (632.8 nm), we rotated the GMR structure azimuthally with the help of a φ-rotation stage as shown in the figure. This gives us additional degrees of freedom (in addition to θ rotation) to tune the GMR peak position precisely^19, 23^.

The laser beam is passed through a spatial filtering setup that contains a 20x microscope objective, a 15 μm pinhole, a collimating lens (focal length of 15 cm) and a 5 mm diameter aperture. The collimated (tested using a collimation tester) and noise free laser beam thus obtained is then allowed to follow the MZI setup. The reflected light from the GMR surface (called as the GMR signal/object beam) interferes with the reference beam through another beam splitter (non-polarizing) generating interference fringes localized at infinity. This interference fringe pattern is recorded by a CMOS camera (DMM 72BUC02-ML USB 2.0 monochrome board) as shown in the figure. We have used a polarizer before the polarizing beam splitter to control and equalize the intensity of the reference & object beam to maximize the contrast of fringe pattern on the CMOS camera.

**Figure 4:**
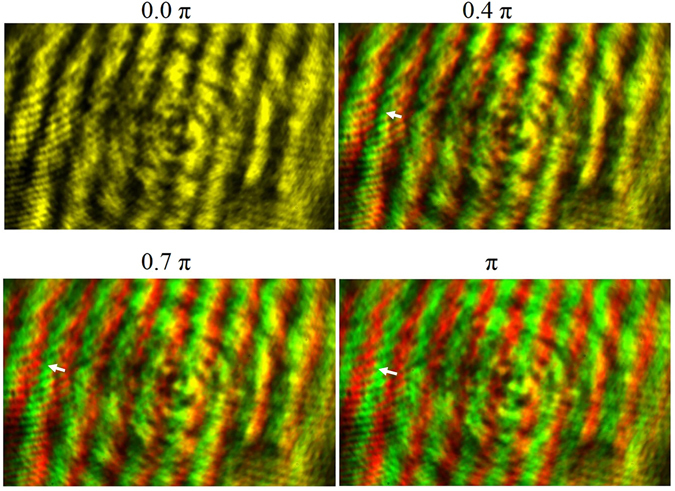
Images of fringe pattern recorded on CMOS. The fringe patterns are for different values of phase shift. The first pattern is when there is no change in phase. For the case of a finite phase shift, the shifted and the initial fringe pattern are indicated by green and red color (false color) respectively. The small white color arrow shows the direction of shift.

As shown in the figure, the GMR sample is fitted to a cuvette that is used to contain the sensing liquid. The process of mounting the GMR sample to the cuvette is provided in the ‘Methods’ section. To increase the RI of the sensing medium, we added sucrose solution drops (by a micropipette) to the reference DI water. The corresponding RI of the final sugar solution is measured by Abbe refractometer. It may be noted that the least count of the Abbe refractometer is 0.0005, but with cautious observation, the reading crosswire may be precisely positioned in between two smallest divisions giving a Δn_min_ = 0.00025. The change in the RI of the sugar solution results in the shift of the interference fringes in accordance with the theory described above. The resulting fringe shift is observed on the CMOS camera and recorded on a PC using ‘IC Capture 2.4’ software. The recorded fringe pattern that shows the spatial displacement of fringes (in terms of pixel values) are further analyzed to find the final phase shift. To have a perception of the fringe shift, we have shown the recorded fringe patterns for different values of phase change (0, 0.4π, 0.7π & π) in Fig. 4.

When there is a finite phase change in the GMR signal, the fringe pattern shifts accordingly and to distinguish the two patterns in the figure, we have indicated the shifted and the original fringe pattern by red and green color (false color) respectively. A complete phase shift of 2π corresponds to a spatial displacement of one bright fringe to the next bright one. In this context it is to be noted that the fringe widths in all these separate cases of measurement are kept equal. However, the sensitivity of detection can be tuned by varying the fringe width. If the fringe width is kept larger, then a phase shift of π covers more number of pixels (n) on the CMOS which corresponds to a higher resolution (π/n).

**Figure 5:**
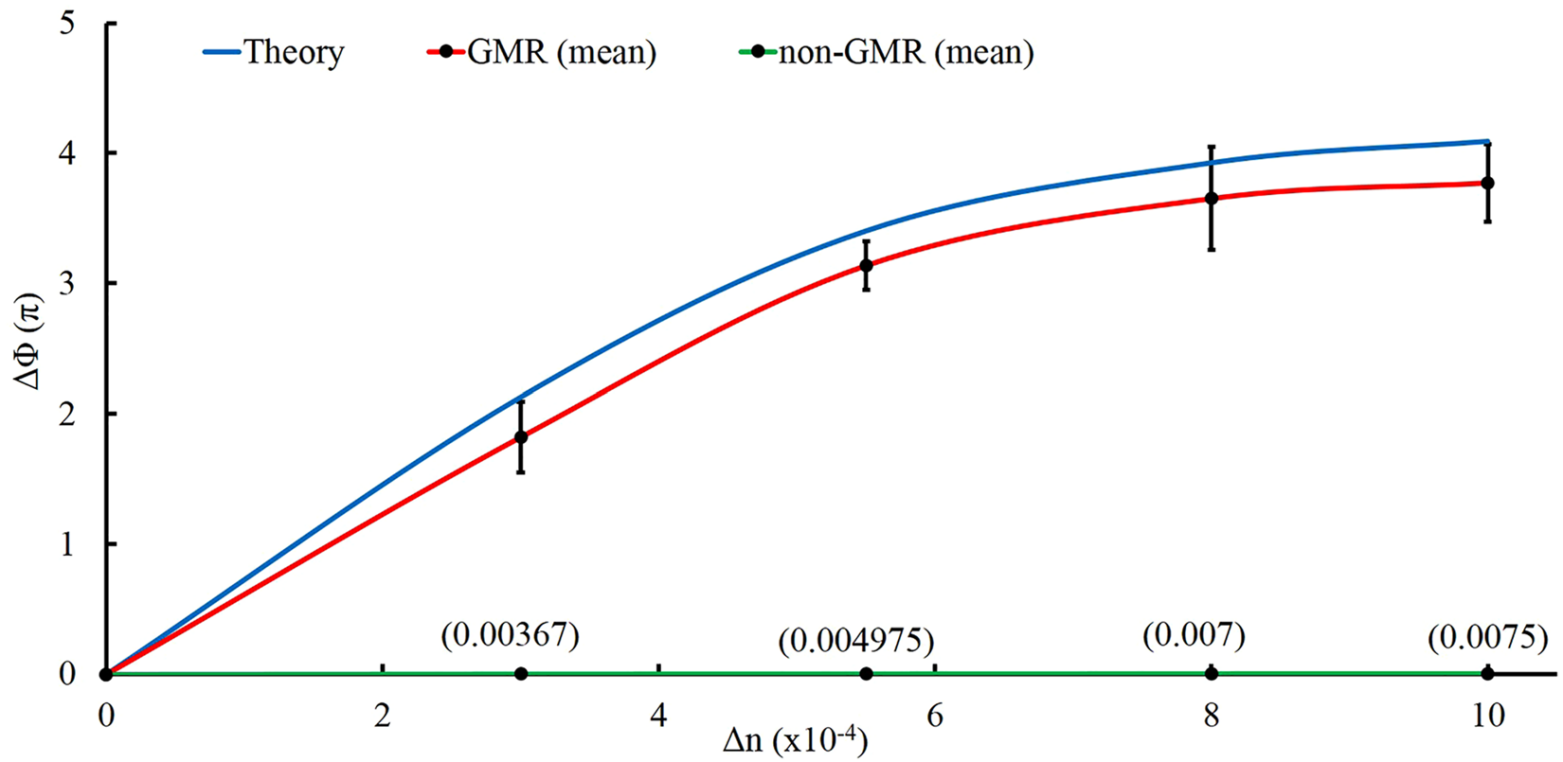
∆Φ vs. ∆n curve. Plot of phase shift (∆Φ) vs. RI change (∆n) of the sensing medium for the GMR as well as non GMR signal. For the GMR signal both the numerically calculated (blue curve) and experimentally obtained (red curve) curve are shown whereas for the non-GMR signal only experimental curve (green curve that lies almost along the horizontal axis) is shown. The error bars are showing the standard deviations (SD) of the data of many measurements.

In Fig. 5, the phase shift (∆Φ) is plotted vs. RI change (∆n) of the sensing liquid. Both experimentally measured and numerically calculated phase shifts are shown in this figure. It is to be noted that the experimental curve (red curve) shown in this figure represents the mean of measurements (the measurement data are shown in Table 1) performed over several times and the error bars are showing the standard deviations (SD) of these data. The obtained values of the SD are reasonably small and the reason of those deviations can be due to the change of surrounding temperature (as the experiments were performed on different days). It can also be inferred from the figure that, the dependency of ∆Φ on ∆n is not linear for higher RI change^11^. Thus considering the linear portion of the above mean curve the sensitivity can be obtained as follows. A change in RI from 1.3352 to 1.3355 leads to a phase shift of 1.8225π that refers to a sensitivity of 0.608π per 10^−4^ RI change which is more than 100 times higher than the already reported MZI based sensor^12, 24, 25^. We have compared the sensitivity of the sensor at GMR position with the non-GMR position by offsetting the GMR device from the resonance condition by rotating it azimuthally. The obtained result which is the experimental mean of many measurements is shown (Fig. 5) as a green colored straight line (labeled as non-GMR) that lies almost along the horizontal axis, since the observed phase change values are too small. The complete experimental data are shown in Table 1. The maximum ∆Φ that is achieved for the non-GMR case is less than 0.1π for a ∆n value that is even six times higher than the previous case. To distinguish the curve from the horizontal axis, we have indicated the ∆Φ values at the corresponding ∆n positions of the plot. Thus the high sensitivity of the sensor is a result of the GMR signal only, whose phase varies abruptly around the resonance wavelength. It is to be noted here that, the FWHM of the GMR peak decides the range of detection for which the above relation between the phase shift and RI change remains linear. Thus the current setup may have limitation to some extent for a broad range of RI change. However in this work, we have solely emphasized on the detection of the smallest possible RI change and not for a broader range.

**Table 1:** Experimentally measured phase change with respect to refractive index change for both GMR & non-GMR position.

No.	Measured RI of sensing liquid	Change in RI (×10^−4^)*	Observed phase shift (π)				Mean shift (π)	SD (π)
			Set 1	Set 2	Set 3	Set 4		
GMR condition								
1	1.3355	3	1.70	1.98	2.16	1.45	1.8225	0.2704
2	1.33575	5.5	3.10	3.35	3.25	2.85	3.1375	0.1883
3	1.336	8	4.16	3.85	3.50	3.10	3.6525	0.3953
4	1.3362	10	4.40	3.96	3.48	3.25	3.7725	0.2958
non-GMR condition								
1	1.33575	5.5	0.000	0.000	0.000	0.000	0.0000	0.0000
2	1.337	18	0.010	0.006	0.008	0.007	0.0076	0.0017
3	1.3375	23	0.045	0.034	0.045	0.055	0.0448	0.0074
4	1.3385	33	0.065	0.073	0.067	0.065	0.0675	0.0031
5	1.341	58	0.090	0.095	0.090	0.085	0.0900	0.0035

*The measured changes are with respect to the reference DI water of RI 1.3352.

From the fringe width and the number of pixels occupied by it, we have calculated (as per the procedure given in the ‘Methods’ section) the minimum detectable phase shift corresponding to a single pixel which comes out to be around (1.94 × 10^−3^)π. Following our obtained sensitivity of 0.608π per 10^−4^ RI change, a phase shift of (1.94 × 10^−3^)π corresponds to a RI change of 3.43 × 10^−7^ RIU. This is the limit of detection of our setup. In this context it should be mentioned that, the limit of detection obtained by our method is quite low. Although not attempted here, following a RI change of ∆n ≈ 10^−6^ for the case of Ar & N_2_ mixture as mentioned in ref. 26, our method can easily detect such a small change in RI and thus efficiently be applied for gas sensing as well.

## Conclusion

In summary, we have demonstrated an experimental method to measure the phase shift of the GMR signal due to RI change of the sensing medium using MZI configuration. We have applied the azimuthal rotation technique to our GMR sample to precisely tune the GMR signal wavelength to our LASER wavelength (632.8 nm). The GMR signal is interfered with a reference signal to produce fringe pattern on the CMOS camera and analyzed further to measure the phase shift. The sharp jump in the phase of the GMR signal around the resonance wavelength makes the fringe pattern very sensitive to the surrounding RI change. In our setup, we have calculated (from the fringe width) the minimum refractive index change of 3.43 × 10^−7^ RIU with a sensitivity of 0.608π phase change per 10^−4^ RI change which are far better than other reported phase detection techniques. We have proposed a theoretical model to numerically calculate the phase shift of this GMR signal that is in good agreement with the experimental results. Unlike other methods, we don’t need to obtain a phase curve requiring phase measurement for a broad wavelength range or incident angles. Experimentally this technique is quite simple and low cost, in comparison to other methods, for example, wavelength shift detection using expensive spectrometers, phase detection using complex electronic heterodyne interferometers etc. It can have wide range of applications in bio-sensing, gas sensing, contamination sensing, explosives sensing etc. In addition to the above advantages, the proposed technique provides easy setting of the resonance condition just by visualizing the intensity shootout of the laser beam at the resonance position, without requiring any broadband source and spectrometer. There is further scope in exploring the effect of temperature of the analyte on the resultant GMR signal phase.

## Methods

### Simulation of phase curve

The phase curve in Fig. 2 is calculated by employing the finite difference time domain (FDTD) simulation method by Lumerical Solutions^27^. In the simulation, we have used 2D (XY) FDTD simulation with periodic boundary conditions along X-axis and absorbing PML boundary condition along Y-axis. For the calculation of the phase of the GMR signal, we have used the ‘s-parameter analysis group’ of Lumerical with the following simulation parameters: auto non-uniform meshing with minimum mesh step of 0.25 nm, the maximum mesh accuracy of 8, stretched coordinate PML type and sufficient simulation time of 2000 fs.

### Mounting of sample in cuvette

The cuvette used in our experimental setup is specially designed and 3D printed to hold the GMR sample properly. The GMR sample is embedded inside a flexible PDMS block which is adhered to the cuvette with the grating surface facing inwards and in contact with the sensing liquid. This flexible PDMS arrangement makes the GMR sample to be adhered tightly to the cuvette wall and confirms it to be leak proof. The GMR sample surface is so sensitive that even a very small amount of pressure generated during the addition of sucrose solution droplets can lead to uncorrelated fringe shift (that is not due to actual RI change). This unwanted fringe shift is minimized by a specially designed perforated wall embedded inside the cuvette such that while the concentration of the measuring solution is changed by addition of further drops, the sensing surface remains completely unperturbed in terms of pressure. This is further confirmed by adding DI water drops to the reference DI water medium that shows no fringe shift except a little distortion in fringe pattern.

### Calculation of limit of detection

The limit of detection is calculated from the fringe width as follows. The area of detection of the CMOS camera is 6 mm × 4 mm with a pixel size of 2.2 μm × 2.2 μm giving a total number of 5 million pixels. For each bright fringe of width 1.61 mm aligned diagonally (as shown in the Fig. 4) on the CMOS sensor surface, number of pixels associated within the width of such bright region is given by (1.61 mm/3.11 μm) ~517, where 3.11 μm is the diagonal width of each pixel. Thus shifting of a bright fringe to a dark fringe involves a shift of 517 pixels which corresponds to a phase change of π radian. Thus the minimum phase shift obtained by a single pixel comes out to be around (1.94 × 10^−3^)π.

#### Figure 3 schematic design

The schematic of the experimental setup shown in Fig. 3 is drawn using Google SketchUp Make 2017 (available at https://www.sketchup.com/download/all).

## Electronic supplementary material

Supplementary Information
