# Supplementary material for: High sensitivity guided-mode-resonance optical sensor employing phase detection
Source: Sci Rep. 2017 Aug 8;7:7607. doi: 10.1038/s41598-017-07843-z (PMC5548775; doi:10.1038/s41598-017-07843-z)
Supplement: Supplementary file 1 — Supplementary Information [file 41598_2017_7843_MOESM1_ESM.pdf]

High sensitivity guided-mode-resonance optical sensor employing phase detection

Pankaj K. Sahoo, Swagato Sarkar and Joby Joseph*

Photonics Research Lab, Department of Physics, Indian Institute of Technology Delhi, New Delhi, 110016, India.
*joby@physics.iitd.ac.in

Derivation of equation (3):

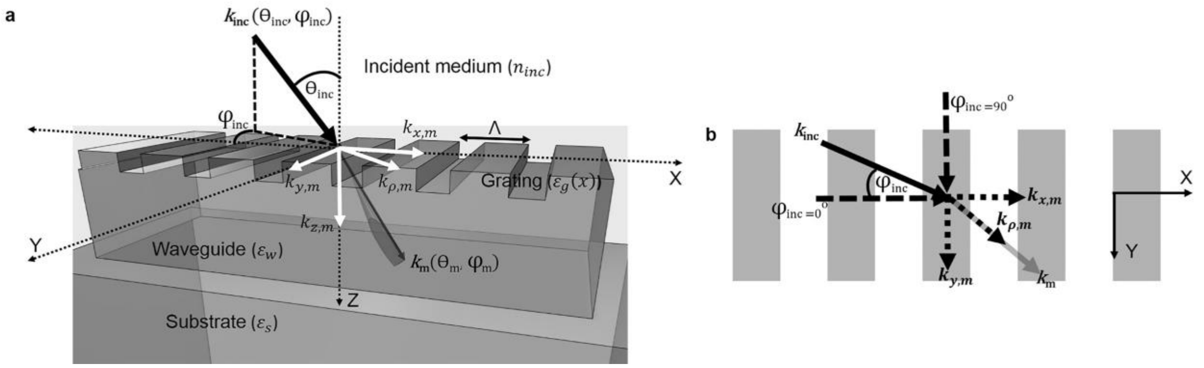

Figure S1. GMR structure under conical mount (a) 3D view (b) Top view

As shown in the 3D view of a GMR structure in Fig. S1 (a), the incident wave vector ($\boldsymbol{k}_{inc}$) in the incidence medium (n_{inc}) is denoted by a set of two spherical coordinate angles (θ_{inc} & φ_{inc}). 'θ_{inc}' is the angle between the incident light beam and the normal (Z-axis) to the grating surface, whereas 'φ_{inc}' is the angle between the plane of incidence with respect to the X-axis. When this GMR structure is rotated azimuthally about the surface normal (Z-axis), the diffracted orders get deviated from the plane of incidence and lie on the surface of a cone. The grating has a grating vector K along X-axis with magnitude $2\pi/\Lambda$, where Λ is the grating period. The average dielectric constant of the grating region is ϵ_{avg}.

As shown in the above figure, under conical mounting (azimuthal rotation), the x, y & z components of the incident wave vector kinc can be expressed in terms of the spherical polar co-ordinates as:

$$\begin{aligned} k_{x,inc} &= k_0 n_{inc} \sin\theta_{inc} \cos\varphi_{inc} &\quad (a)\\ k_{y,inc} &= k_0 n_{inc} \sin\theta_{inc} \sin\varphi_{inc} &\quad (b) \qquad (S1)\\ k_{z,inc} &= k_0 n_{inc} \cos\theta_{inc} &\quad (c) \end{aligned}$$

The x, y & z-components of the diffracted wave are expressed as:

$$\begin{aligned} k_{x,m} &= k_{x,inc} + mK &\quad (a)\\ k_{y,m} &= k_{y,inc} &\quad (b)\\ k_{z,m} &= \sqrt{k^2 - {k_{\rho,m}}^2} &\quad (c) \qquad (S2)\\ \text{with,} \quad k_{\rho,m} &= \sqrt{{k_{x,m}}^2 + {k_{y,m}}^2} &\quad (d) \end{aligned}$$

The propagation constant of the diffracted wave, β_m can be expressed in terms of the incident wave vector ($k_{x,inc}$ & $k_{y,inc}$) as $\beta_m = \sqrt{{k_{x,m}}^2 + {k_{y,m}}^2}$.

Using the value of $k_{x,m}$ from eq. S2 (a), we get

$$\beta_m = \sqrt{\left(k_{x,inc} + mK\right)^2 + {k_{y,inc}}^2} = \sqrt{{k_{x,inc}}^2 + {k_{y,inc}}^2 + 2k_{x,inc} mK + m^2K^2}$$

Expanding the wave vector components in terms of their respective spherical polar co-ordinates from equation (S1), $n_{eff}\left(=\frac{\beta_m}{k_0}\right)$ can be expressed as

$$n_{eff} = \sqrt{(n_{inc}\, sin\,\theta_{inc})^2 + 2n_{inc}\, sin\,\theta_{inc}\, cos\,\varphi_{inc} m\frac{\lambda_0}{\Lambda} + m^2\frac{{\lambda_0}^2}{\Lambda^2}}$$

This proves equation (3).

Image of the GMR sample and AFM data:

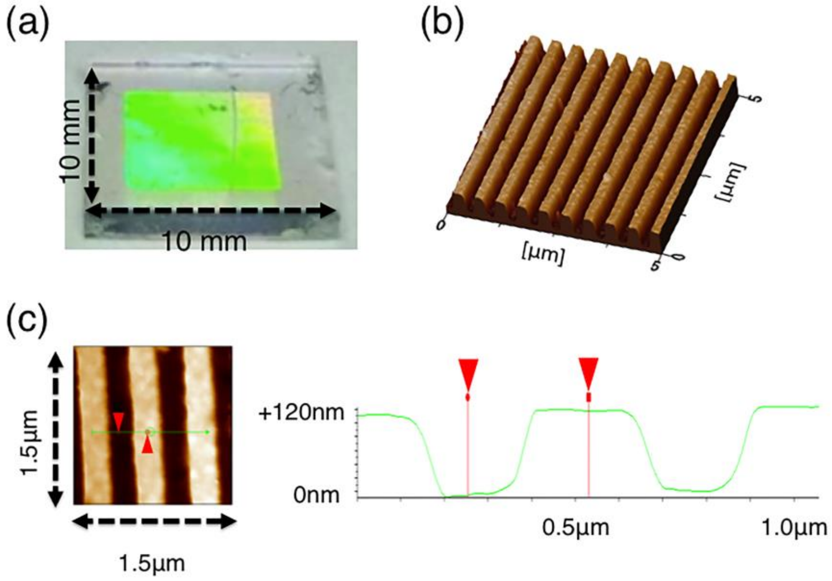

Figure S2. Characterization of the fabricated GMR structure (a) Photograph of the GMR sample that shows a 10mm x 10mm substrate with 6mm x 6mm pattern area (b) AFM topography of the GMR structure (c) AFM measurements show grating depth to be 120 nm.

Image of the actual experimental set up:

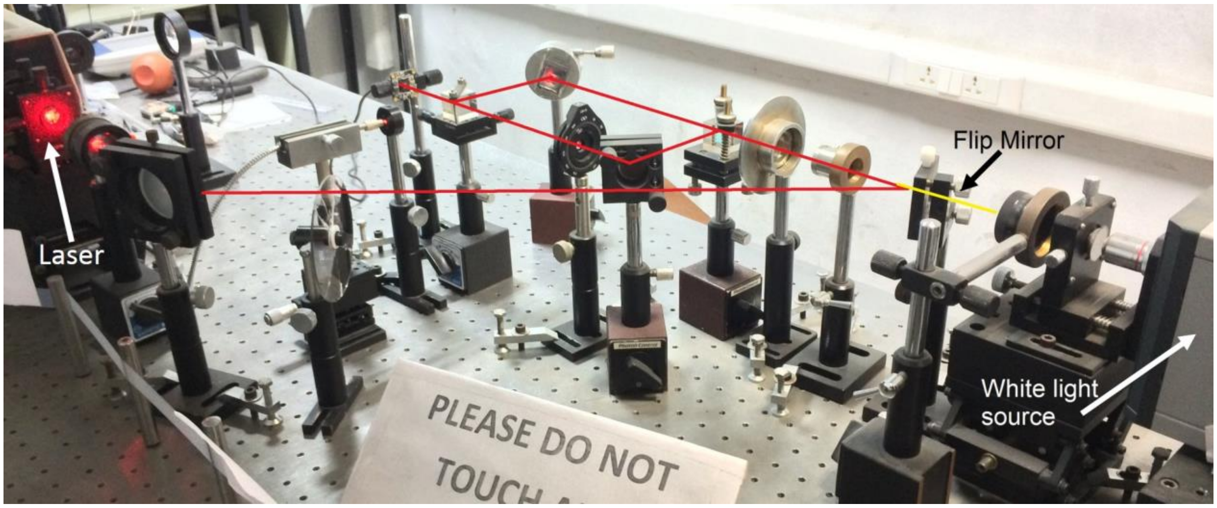

Figure S3. Experimental setup showing the laser and white light source, both following the same MZI path via a flipped mirror.
